# Supplementary material for: Development of a Potential Gallium-68-Labelled Radiotracer Based on DOTA-Curcumin for Colon-Rectal Carcinoma: From Synthesis to In Vivo Studies
Source: Molecules. 2019 Feb 12;24(3):644. doi: 10.3390/molecules24030644 (PMC6384893; doi:10.3390/molecules24030644)
Supplement: Supplementary file 1 [file molecules-24-00644-s001.pdf]

Development of a potential gallium-68 labelled radiotracer based on DOTA-curcumin for colon-rectal carcinoma: from synthesis to in vivo studies.

Giulia Orteca [1], Federica Pisaneschi [2], Sara Rubagotti [3], Tracy W. Liu [2], Giacomo Biagiotti [2], David Piwnica-Worms [2], Michele Iori [3], Pier C. Capponi [3], Erika Ferrari [1,*] and Mattia Asti [3]

[1] Department of Chemical and Geological Sciences, University of Modena and Reggio Emilia, via G. Campi 103, 41125, Modena, Italy
[2] Department of Cancer Systems Imaging, The University of Texas MD Anderson Cancer Center, 1881 East Road, 77054 Houston, Texas, USA.
[3] Radiopharmaceutical Chemistry Unit, Oncologic and High Technologies Department, Azienda Unità Sanitaria Locale-IRCCS, via Amendola 2, 42122, Reggio Emilia, Italy.
[4] Department of Chemistry "Ugo Schiff", Università di Firenze, via della Lastruccia 3-13, 50019, Sesto Fiorentino, Italy.

* Correspondence: erika.ferrari@unimore.it Tel.: +39-059-2058631; fax. +39 059 205 8631

1. List of contents:

- Chemical characterization of ligand and complexes
- Quality controls on radioactive preparations
- Figure S1: ESI-LC-MS analysis of DOTA-C21.
- Figure S2: ^{1}H NMR spectra of DOTA-C21 in D_2O and in MeOD-d_4 at 25 °C.
- Figure S3: ^{13}C NMR spectra of DOTA-C21 in MeOD-d_4 at 25 °C.
- Figure S4: ESI-LC/MS analysis of Ga-DOTA-C21.
- Figure S5: Detailed ESI-LC/MS of one Ga-DOTA-C21 isomer.
- Figure S6: ^{1}H NMR titration of DOTA-C21 with Ga^{3+}.
- Figure S7: Aliphatic region of ^{1}H,^{1}H COSY NMR spectrum of Ga-DOTA-C21 in MeOD-d_4 at 25 °C ([DOTA-C21] = 0.63 mM).
- Figure S8: LC/MS fragmentation experiments on curcumin.
- Figure S9: UV–vis absorption spectra of the system Ga^{3+}/DOTA-C21.
- Figure S10: Fluorescence emission spectra of the system Ga^{3+}/DOTA-C21.
- Figure S11: TLC analyses of ^{68}Ga-DOTA-C21 prepared by using the post processing method.
- Figure S12: Average biodistribution of ^{68}Ga-DOTA-C21 extracted from micro-PET data at various time points post *i.p.* and *i.v.* injection. Correlation between T/M ratio and tumour size.
- Figure S13: In *vivo* stability of ^{68}Ga-DOTA-C21 in murine blood samples.

2. Chemical characterization of ligand and complexes

All chemicals were reagent grade and used without further purification unless otherwise specified.

2.1. ESI-LC-MS analyses

Liquid chromatography/mass spectrometry (LC/MS) was performed on Agilent 6300 Ion Trap LC/MS System equipped with an electrospray ionisation (ESI) interface. The compounds were separated using Agilent Zorbax SB C18 30x2.1mm, 3.5µm; the blanks were MilliQ water. Eluent phase: A = H_2O (formic acid 0.1%), pump B = CH_3CN (formic acid 0.1%), gradient: 10–100% of B in 5 min, 0.3 mL/min. Mass spectra were recorded in alternate modality, using a scan range between 100 and 1500 m/z. High-purity nitrogen was used as nebuliser and drying gas. The nitrogen drying gas was kept at a constant flow rate of 10 L/min and heated to 350 °C. The nebuliser gas pressure was 32 psi and the capillary voltage was 3.5 kV. MS spectra of the ligand (concentration of 11 ppm, *m*/*z* = 798.4 $[M+H]^+$) and gallium metal complex (concentration of 12 ppm, M:L 1:1, *m*/*z* = 864.3 $[M\text{-}^{69}Ga+H]^+$, 866.3 $[M\text{-}^{71}Ga+H]^+$) were recorded.

2.2. NMR Spectroscopy

NMR spectra were recorded by means of FT-NMR AVANCE III HD 600 MHz spectrometer (Bruker Biospin) equipped with a CryoProbe BBO H&F 5mm. Nominal frequencies were 150.90 MHz for ^{13}C, and 600.13 MHz for ^1H. NMR sample was prepared dissolving 0.3 mg of free ligand in 0.6 mL of D_2O or MeOD-d4. Synthesis of Ga-DOTA-C21 complexes were obtained *in situ*, by adding to a 0.3 mg DOTA-C21 solution (0.6 mL) the proper amount of $Ga(NO_3)_3{\cdot}9H_2O$ solution to reach a 1:1 metal to ligand molar ratio. Spectra were registered at room temperature after few minutes from addition.

2.3. Fragmentation experiments

The experiments were performed on the same Liquid chromatography/mass spectrometry (LC/MS) instrument described here before. The LC measurements were performed using Agilent Zorbax SB C18 30x2.1mm, 3.5µm. The Eluent phases were H_2O (0.1% formic acid) and CH_3CN (0.1% formic acid), the gradient used was 10–100% of CH_3CN (0.1% formic acid) in 6 minutes with a flow of 0.3 mL/min. Mass spectra were obtained with a soft ionization method, recording in alternate modality, and using a scan range between 100 and 1500 m/z. High-purity nitrogen was used as nebulizer and drying gas. Drying gas was at a constant flow rate of 10 L/min, heated to 150 °C. Nebulizer gas pressure was 32 psi and the capillary voltage was 1.5 kV. The fragmentation experiments were carried out still with soft ionization procedure but in positive modality, selecting the target mass to fragment in ionic trap until MS/MS^3 spectra. Sample of cold complex was prepared from a solution of 1000 ppm in 0.4 M Ammonium Acetate buffer (pH 4.4) and diluted in order to inject 5 µL of 50 ppm complex solution. To validate the Ga-DOTA-C21 complex fragmentation, curcumin fragmentation experiment was performed in same conditions, preparing the starting solution of 1000 ppm in methanol, then diluting the solution in the same buffer to 50 ppm.

2.4. UV-visible experiments

UV–visible absorption measurements were performed using a Varian Cary 100 spectrophotometer in the range 200-600 nm. All measurements were performed at 25 °C. The titration was performed using 25 mL of $3{\cdot}10^{-6}$ M ligand solution in PBS buffer (pH 7.14), increasing amount of $1.25{\cdot}10^{-3}$ M $Ga(NO_3)_3{\cdot}9H_2O$ solution were added up to the 1:1 molar ratio (from 5 µL to 60 µL). In these conditions volume variation was negligible. Spectra were acquired few minutes after each addition.

2.5. Fluorescence experiments

Fluorescence experiments were carried out on a Spex Jobin-Yvon Fluoromax-3 spectrofluorometer. Emission spectra were obtained using as excitation λ 410 nm and recorded between 750 nm and 425 nm. All

measurements were performed at 25 °C. Titration was performed using the same procedure described for UV-visible studies.

3. Quality controls on radioactive preparations

Assessment of the completion of the radiolabeling reaction were performed by UHPLC (Waters, Milan, Italy) equipped with a Berthold radio-detector and an TUV Acquity UV-detector, and by Radio-TLC analysis (Elysia, Liege, Belgium). UHPLC were carried out on a BEH C-18 1.7 m 21x150 mm column at a flow rate of 0.35 mL/min using A: CH_3CN and B: 0.1% vol/vol TFA water solution as mobile phase with the following gradient: 0-1 min 10 % A, 1-8 min 10-95 % A. The wavelength of the UV detector was set to 254 nm and the column temperature was fixed to 30 °C. In order to identify the chromatographic peaks during the analysis, free $^{68}Ga^{3+}$ and ^{68}Ga-hydrolyzed products were prepared as reference standard. TLC analyses were performed by using two system: i) RP-TLC plates were developed in 0.1 M citrate buffer (pH 4) ii) ITLC-SG plates were developed in ammonium acetate 1M / MeOH 1:1 v/v solution, using a flatbed-imaging scanner.

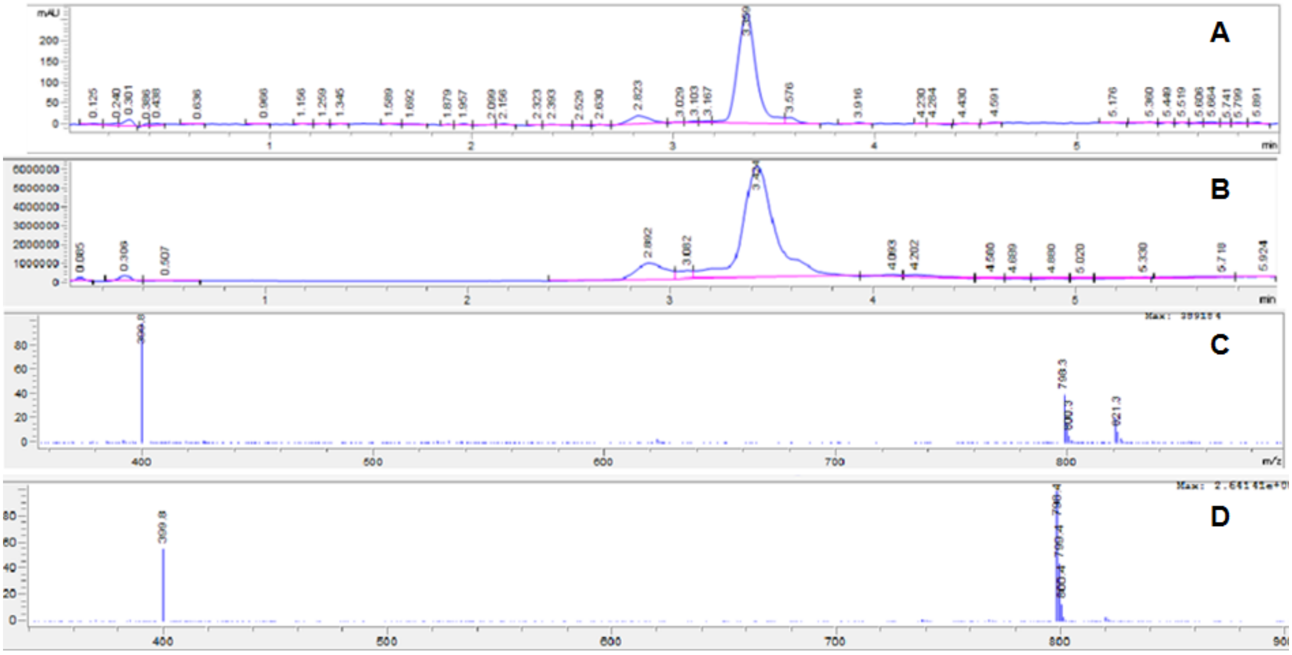

Figure S1. ESI-LC/MS analysis of DOTA-C21. HPLC chromatogram on UV 254 nm detector (**A**) HPLC chromatogram on MSD1 detector (**B**). MS spectrum of the 2.885 minute region (**C**). MS spectrum of the 3.412 minute (**D**).

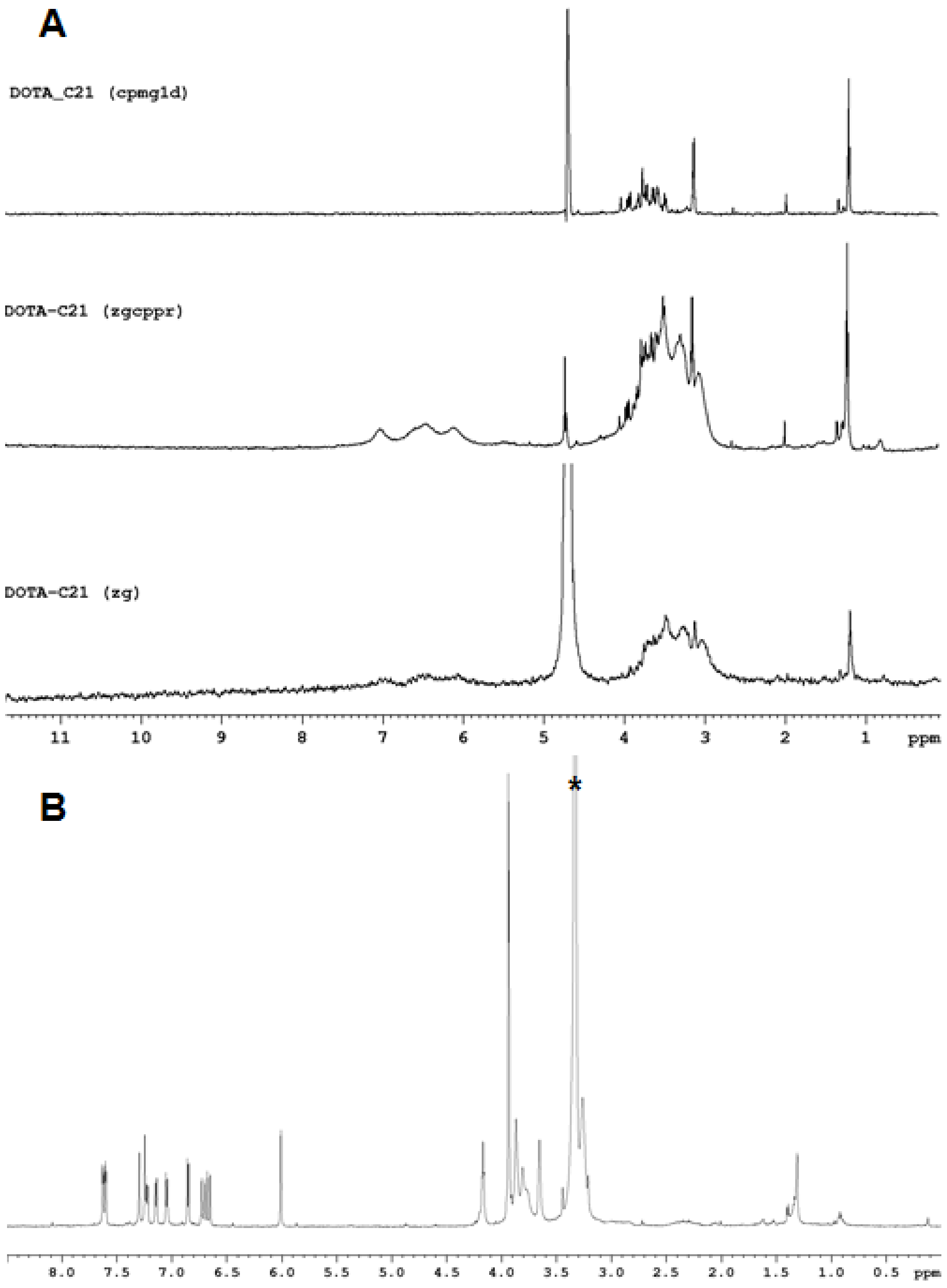

Figure S2. ^{1}H NMR spectra of DOTA-C21 in D_2O at 25 °C obtained by three different pulse sequences according to Bruker library: water-suppression using pre-saturation pulses (*zgcppr*), selection of sharp signals by application of Carr-Purcell-Meiboom-Gill (CPMG) pulse program (*cpmg1d*) and basic 90° pulse sequence (*zg*), (**A**). ^{1}H NMR spectrum of DOTA-C21 in MeOD-d_4 at 25 °C obtained by *zgcppr* pulse sequence (* = residual solvent peak) (**B**).

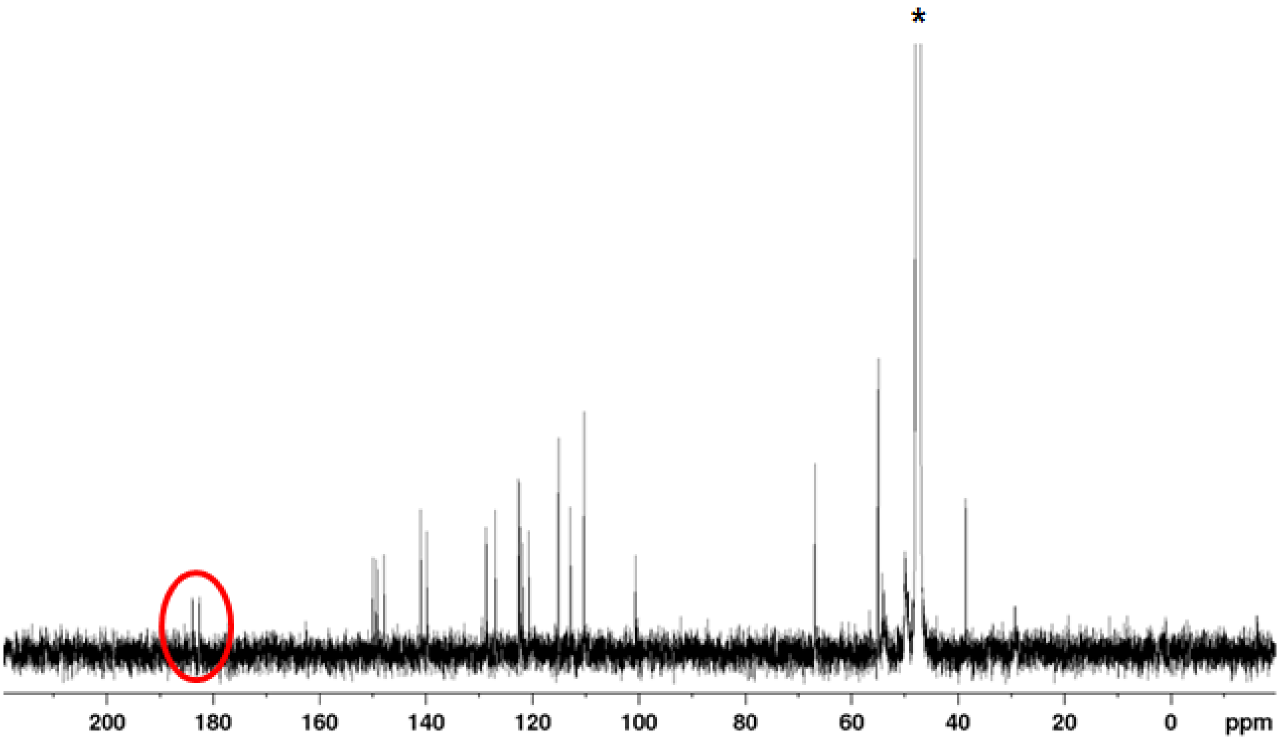

Figure S3. ^{13}C NMR spectrum of DOTA-C21 in MeOD-d_4 at 25 °C (* residual solvent peak). Red ellipsis highlights the non-equivalence of enol- and keto- ^{13}C chemical shift.

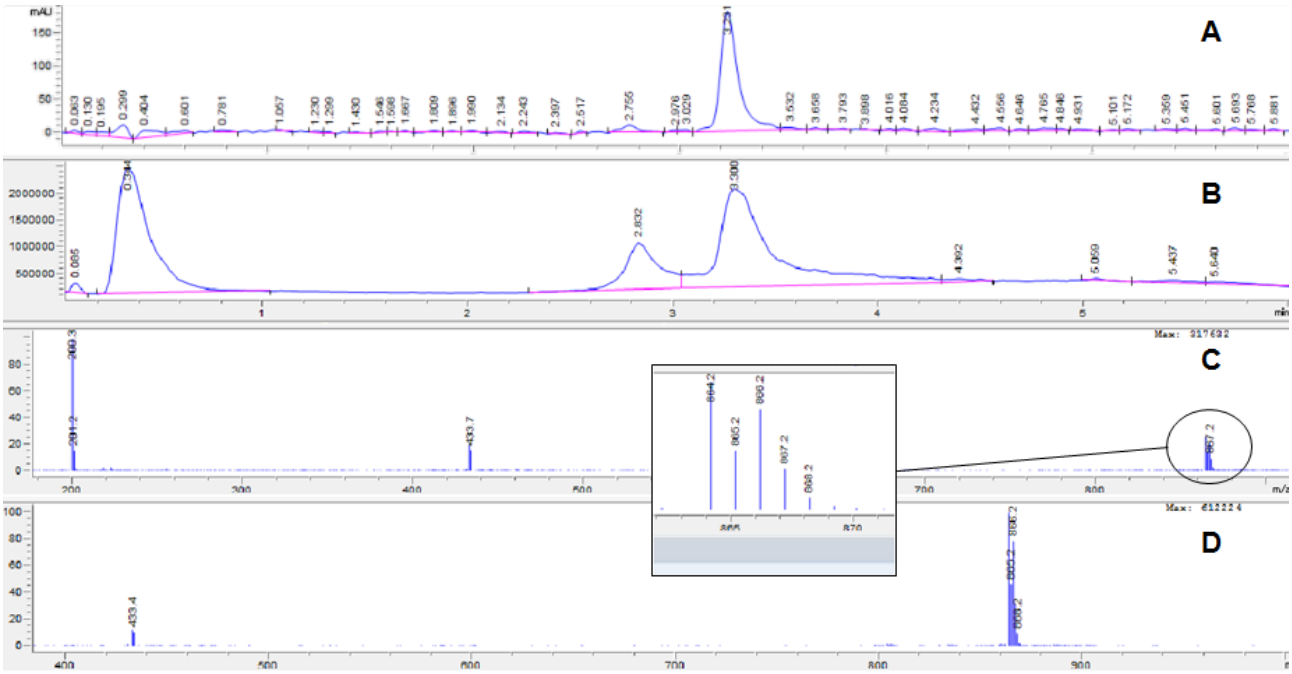

Figure S4. ESI-LC/MS analysis of Ga-DOTA-C21. HPLC chromatogram on UV 254 nm detector (**A**) HPLC chromatogram on MSD1 detector (**B**). MS spectrum of the 2.808 minute region (**C**). MS spectrum of the 3.297 minute (**D**). Gallium isotopic pattern is magnified in the square

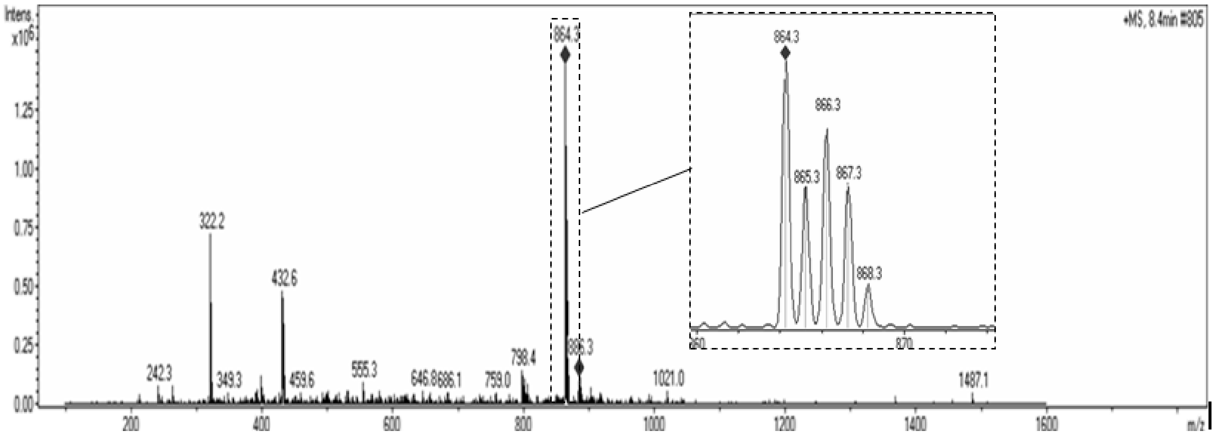

Figure S5. Detailed ESI-LC/MS spectrum of one of the two Ga-DOTA-C21 isomers with gallium isotopic pattern magnified in dashed square.

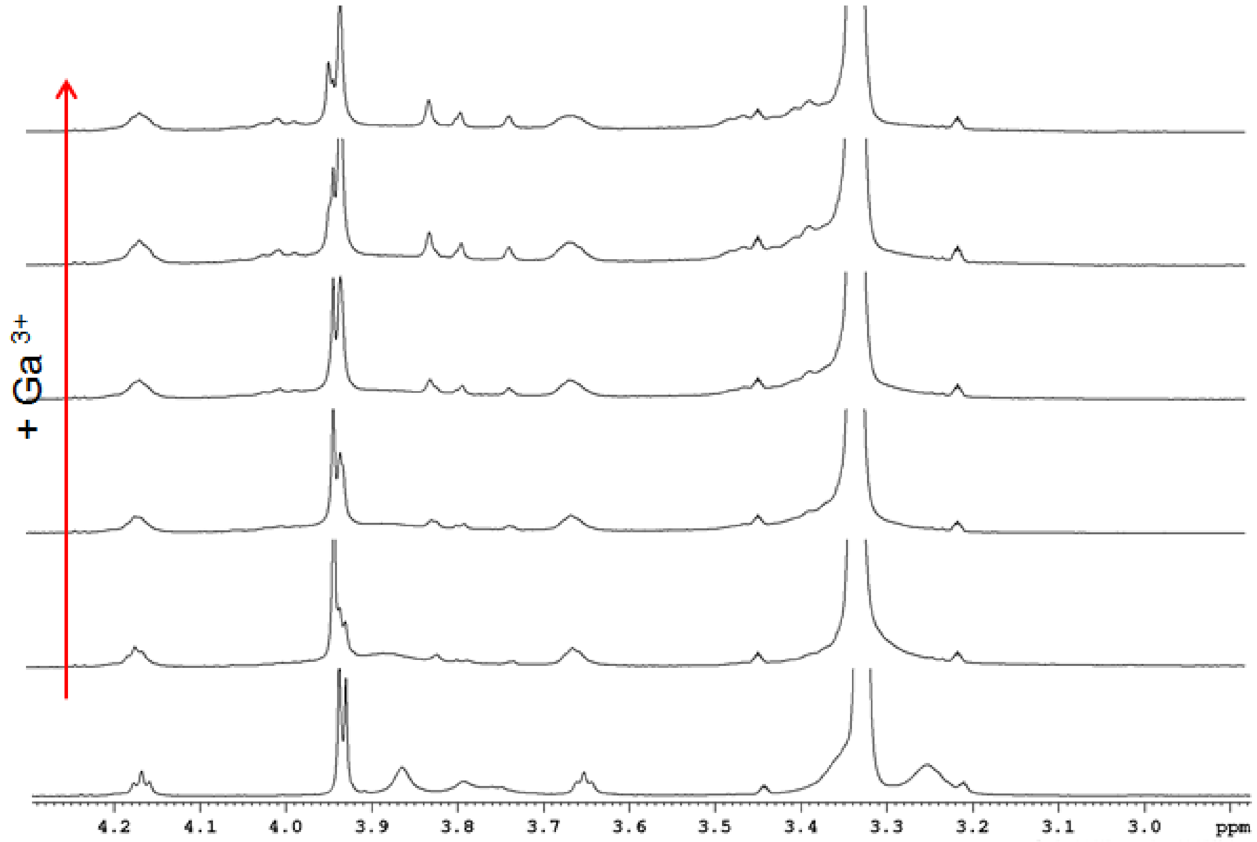

Figure S6. ^{1}H NMR titration of DOTA-C21 with Ga^{3+} solution, from bottom to top: free chelator and incrementing addition of Ga^{3+} solution, up to 1:1 metal-to-ligand molar ratio (top). All the spectra were acquired in MeOD-d_4 at 25 °C ([DOTA-C21] = 0.63 mM).

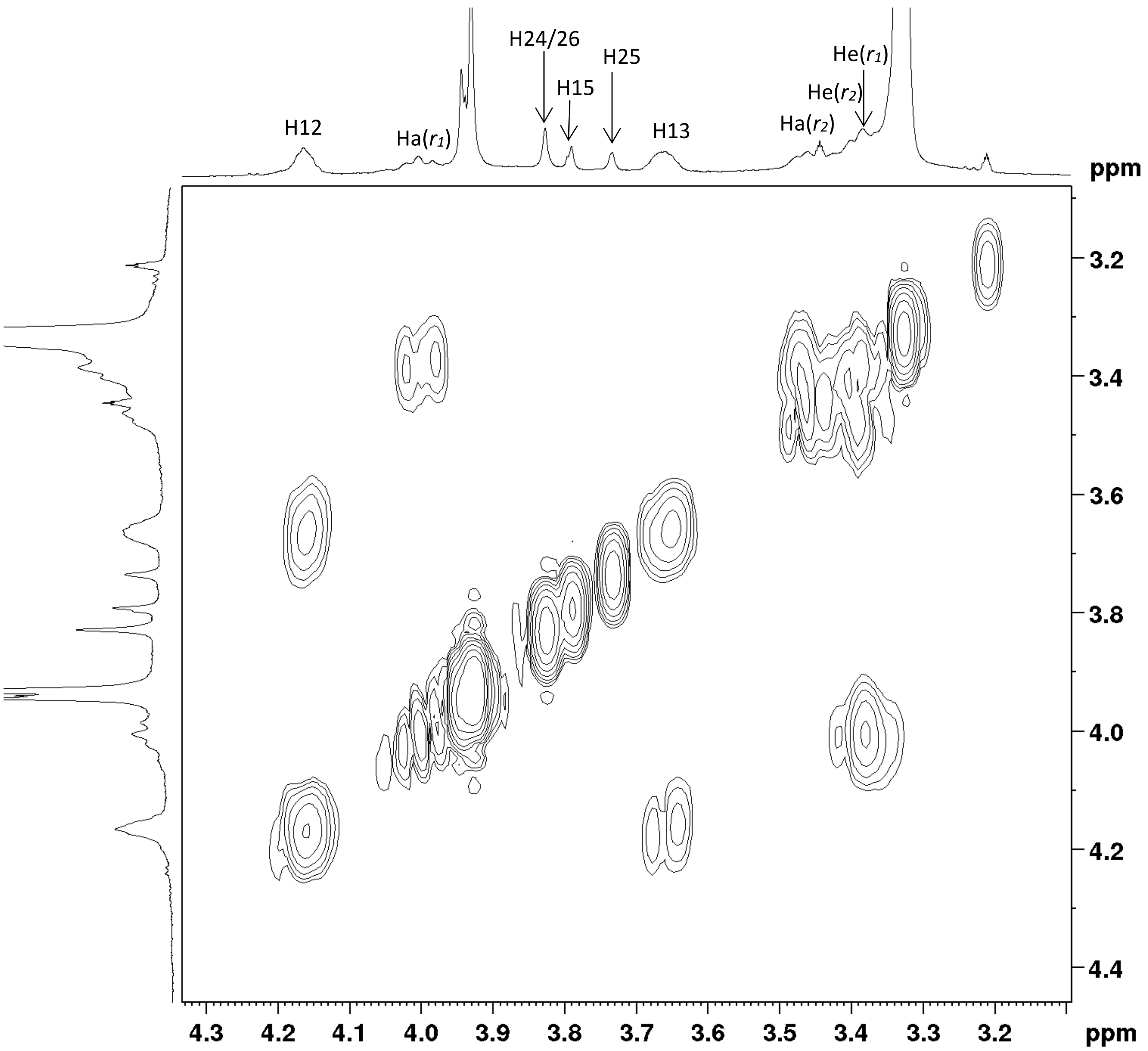

Figure S7. Aliphatic region of ^{1}H,^{1}H COSY NMR spectrum of Ga-DOTA-C21 in MeOD-d_4 at 25 °C ([DOTA-C21] = 0.63 mM).

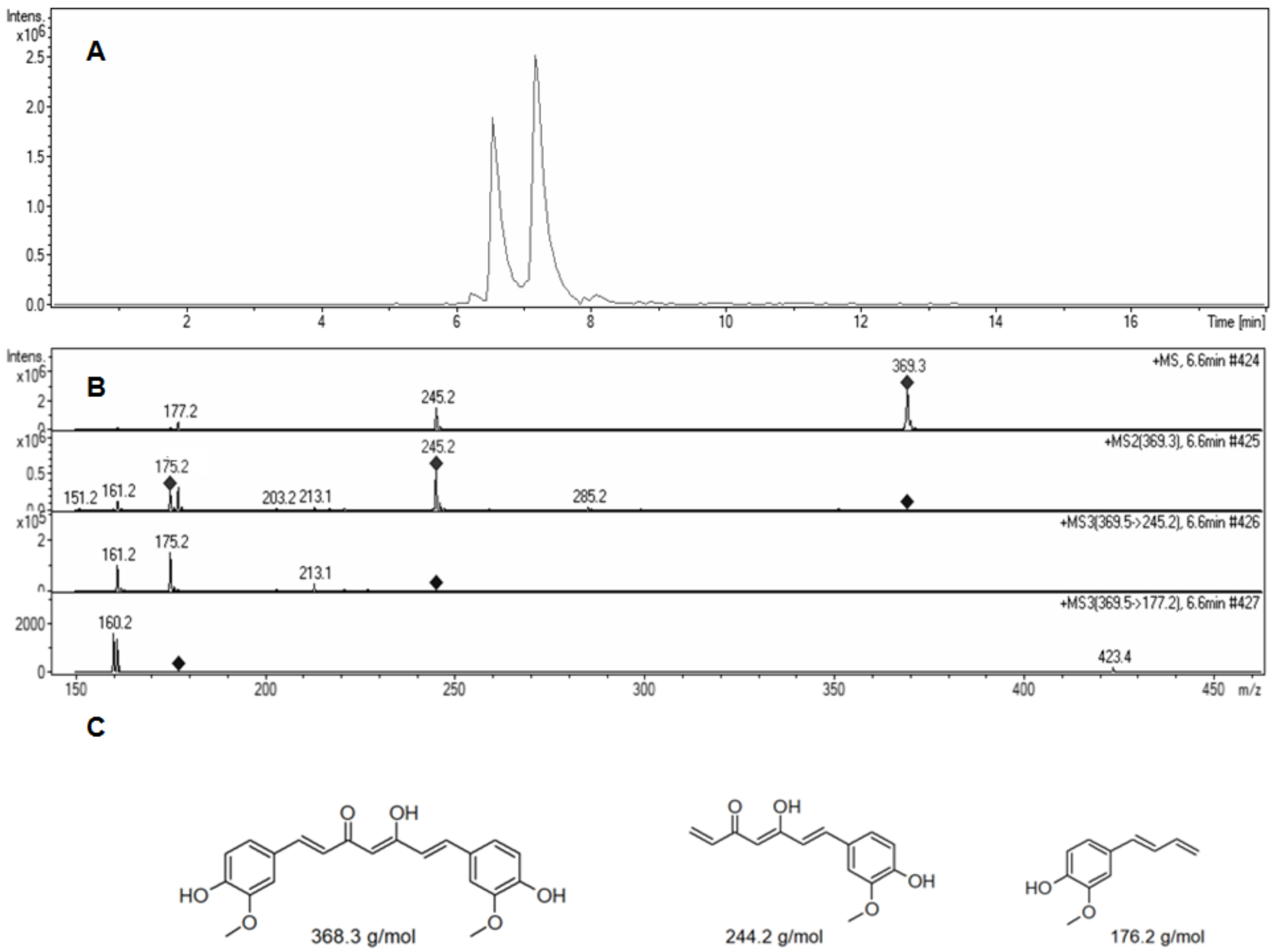

Figure S8. LC/MS fragmentation experiments on curcumin. HPLC-MS chromatogram (**A**) Fragmentation pathway of the *m/z* = 369.5 $[M+H^+]$ ion, corresponding to the peak at retention time 6.6 minutes (**B**). Molecular fragments attributed to the *m/z* = 369.5 $[M+H]^+$ pattern (**C**).

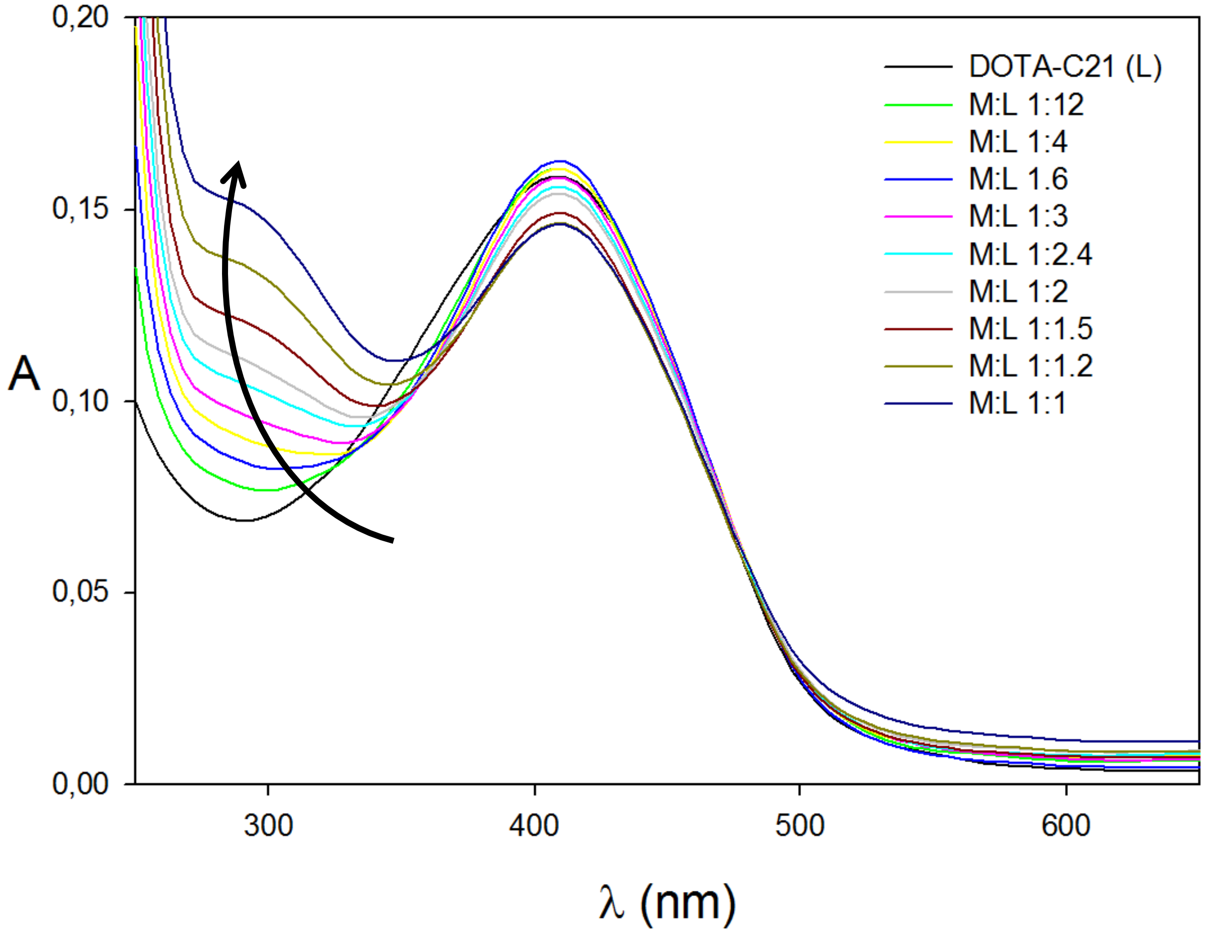

Fig. S9 UV-vis spectra of DOTA-C21 in PBS solution (pH 7.4) upon increasing addition of Ga^{3+}: from free ligand (black spectrum) up to metal to ligand 1:1 molar ratio (dark-blue spectrum) ([DOTA-C21] = 3 μM). Black arrow highlights the change in the spectrum at increasing Ga^{3+} concentration.

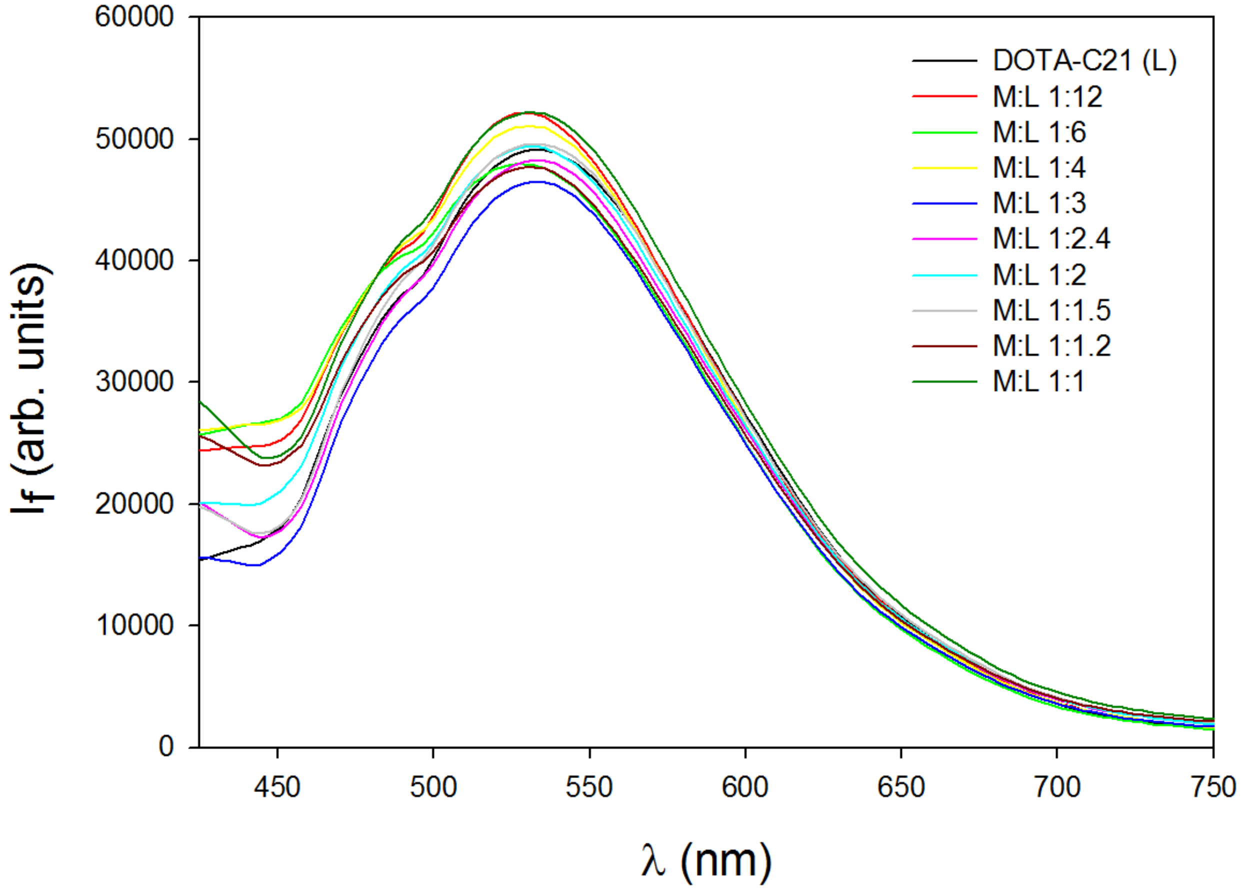

Figure S10. Fluorescence emission spectra of the system Ga^{3+}/DOTA-C21 from free ligand (black spectrum) up to metal to ligand 1:1 molar ratio (dark green spectrum) (λ_{ex} 410 nm, [DOTA-C21]=3 μM).

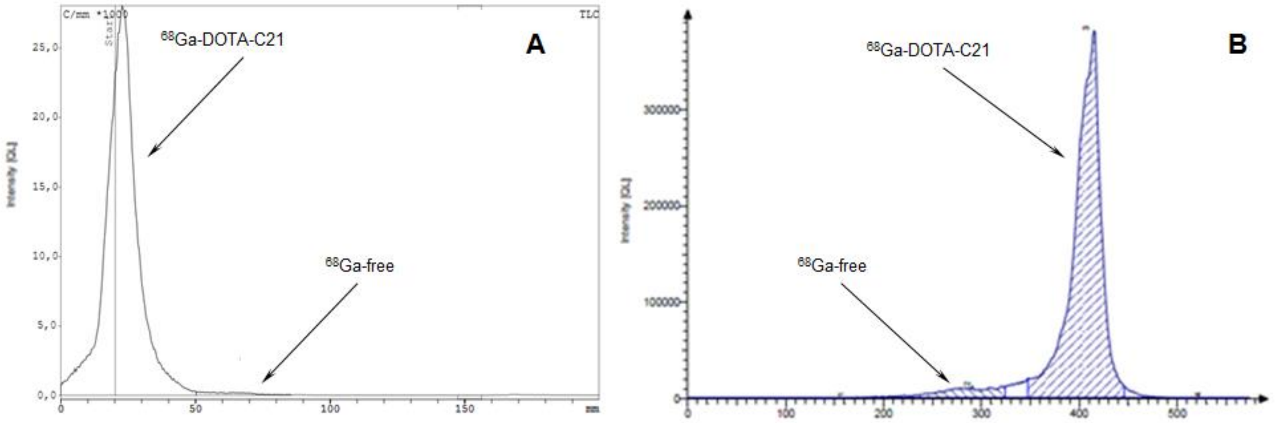

Figure S11. Representative radio-TLC analyses of Ga-DOTA-C21 prepared by using the post processing method after 15 minutes of heating at 95 °C. RP-TLC plates scan developed in 0.1 M citrate buffer (pH 4) (**A**) and ITLC-SG scan developed in ammonium acetate 1M / MeOH 1:1 v/v solution (**B**).

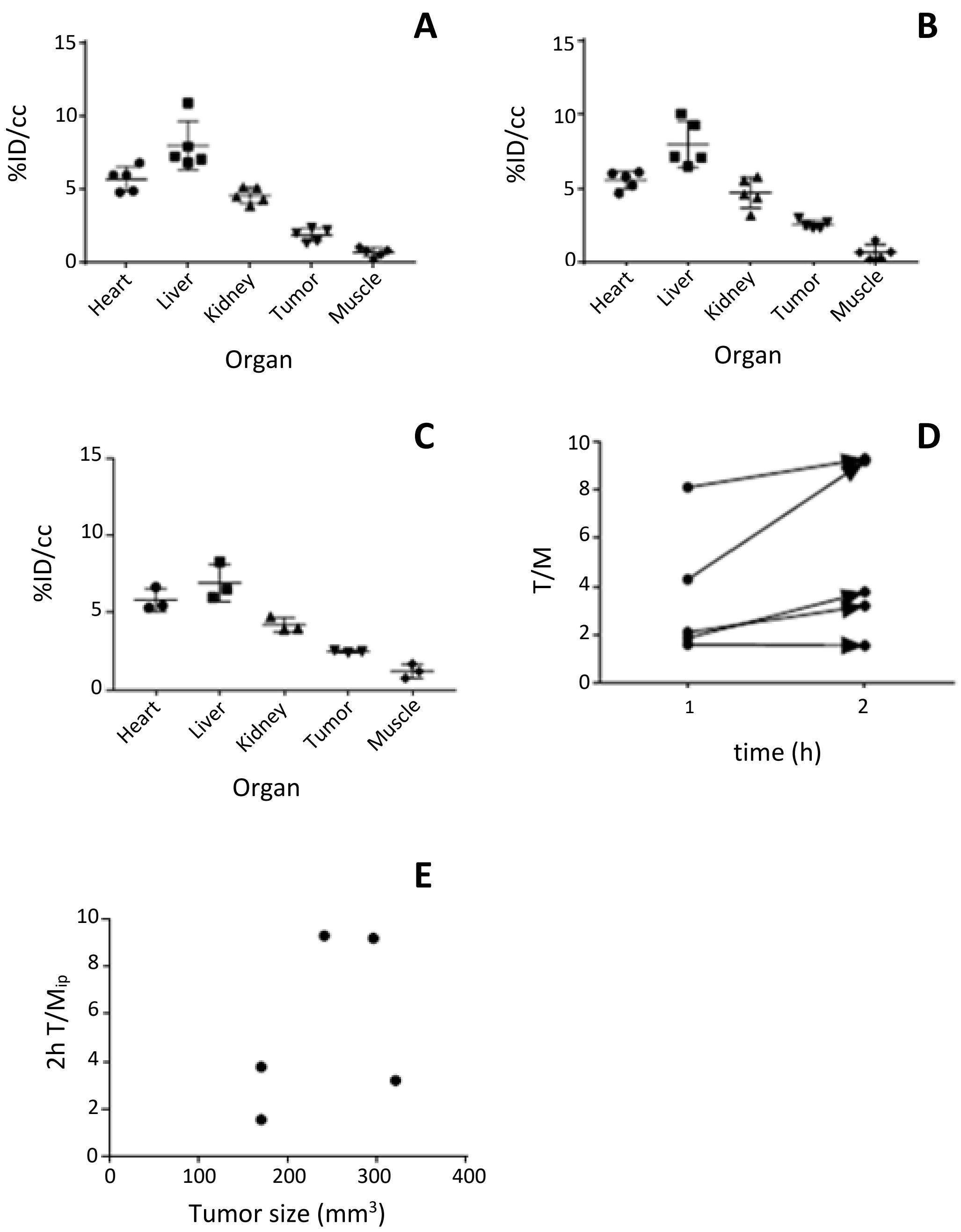

Figure S12. Average biodistribution of ^{68}Ga-DOTA-C21 extracted from micro-PET data at 1h (**A**) and 2h (**B**) post *i.p.* injection (n = 5, mean ± SD). Average biodistribution of ^{68}Ga-DOTA-C21 extracted from micro-PET data at 1h post *i.v.* injection (n = 3, mean ± SD (**C**). Variation of the T/M ratio with time for each individual mouse (**D**). Correlation between T/M ratio and tumour size (mm^3) (**E**).

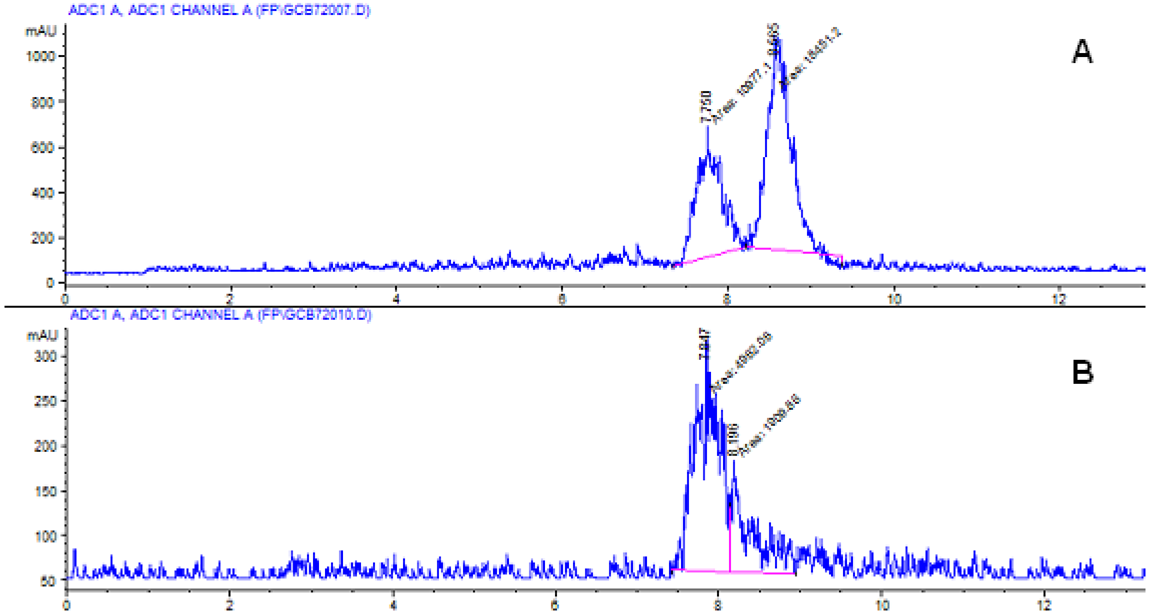

Figure S13. In *vivo* stability of ^{68}Ga-DOTA-C21 in blood samples at the injection time (**A**) and 90 minutes *post* injection (**B**).
